# Supplementary material for: NSAID use is not associated with Parkinson’s disease incidence: A Norwegian Prescription Database study
Source: PLoS One. 2021 Sep 7;16(9):e0256602. doi: 10.1371/journal.pone.0256602 (PMC8423296; doi:10.1371/journal.pone.0256602)
Supplement: S1 Table — (DOCX) [file pone.0256602.s001.docx]

**S1 Table.**

**Demographics and descriptive statistics for Norwegian Prescription Registry**

**Groups used in Time-dependent Cox Regression Analysis with 90DDD^a^ Threshold**

| Demographics 90DDD Threshold: | Diclofenac^b^ | Diclofenac + ASA^c^ |  | Ibuprofen^d^ | Ibuprofen + ASA^e^ |  |
| --- | --- | --- | --- | --- | --- | --- |
| Total Number: | 126.045 | 85.859 |  | 76.009 | 49.013 |  |
| Sex (Male %): | 38% | 47% |  | 30% | 38% |  |
| Follow-up time mean months (SD) | 79.9  (42.3) | 87.0  (41.3) |  | 76.2  (45.6) | 83.7  (44.4) |  |
| Total observation time (years)^f^ | 1.223.387 | 773.847 |  | 581.392 | 398.426 |  |
| Total Immortal time(years)^g^ | 224.822 | 150.532 |  | 98.780 | 56.667 |  |
| Mean age  (SD) | 70.0  (8.8) | 75.1  (9.3) |  | 69.1  (9.3) | 74.7  (9.8) |  |
| Age 50-55 (%) | 948  (1%) | 96  (<1%) |  | 1.721  (2%) | 233  (<1%) |  |
| Age 55-60 (%) | 8.468  (7%) | 1.578  (2%) |  | 8.048  (10%) | 1.632  (3%) |  |
| Age 60-65 (%) | 29.083  (23%) | 9.609  (11%) |  | 17.551  (23%) | 6.032  (12%) |  |
| Age 65-70 (%) | 31.261  (25%) | 15.780  (18%) |  | 17.509  (23%) | 8.930  (18%) |  |
| Age 70-75 (%) | 23.493  (19%) | 17.510  (20%) |  | 12.789  (17%) | 9.411  (19%) |  |
| Age 75-80 (%) | 13.538  (11%) | 13.822  (16%) |  | 7.271  (10%) | 7.285  (15%) |  |
| Age > 80 (%) | 19.254  (15%) | 27.464  (32%) |  | 11.120  (15%) | 15.490  (32%) |  |
| Median cumulative DDD exposure^h^ | 200 | 220 |  | 250 | 250 |  |
| Deaths (%) | 11.8% | 20.6% |  | 12.8% | 22% |  |
| PD Incidence number | 744 | 626 |  | 405 | 313 |  |
|  |  |  |  |  |  |  |
| Demographics 90DDD Threshold | **Naproxen^i^** | **Naproxen + ASA^j^** |  |  |  |  |
| Total Number | 98.324 | 58.679 |  |  |  |  |
| Sex (Male %): | 36% | 46.7% |  |  |  |  |
| Follow-up time mean months (SD) | 46.6  (41.6) | 58.3  (45.2) |  |  |  |  |
| Total observation time (years)^f^ | 474.470 | 343.136 |  |  |  |  |
| Total Immortal time(years)^g^ | 92.870 | 58.294 |  |  |  |  |
| Mean age  (SD) | 67.0  (9.7) | 73.9  (10.1) |  |  |  |  |
| Age 50-55 (%) | 6.661  (7%) | 849  (1%) |  |  |  |  |
| Age 55-60 (%) | 17.544  (18%) | 3.457  (6%) |  |  |  |  |
| Age 60-65 (%) | 20.862  (21%) | 6.997  (12%) |  |  |  |  |
| Age 65-70 (%) | 18.450  (19%) | 9.733  (17%) |  |  |  |  |
| Age 70-75 (%) | 14.522  (15%) | 11.021  (19%) |  |  |  |  |
| Age 75-80 (%) | 8.671  (9%) | 9.185  (16%) |  |  |  |  |
| Age>80 (%) | 11.614  (12%) | 17.437  (30%) |  |  |  |  |
| Median cumulative DDD exposure^h^ | 200 | 210 |  |  |  |  |
| Deaths (%) | 6.8% | 15.9% |  |  |  |  |
| PD Incidence number | 297 | 259 |  |  |  |  |

a)Minimum of 365 cumulative defined daily dose of NSAID (diclofenac, ibuprofen or Naproxen) exposure during follow up. b) Diclofenac users with less than 90 cumulative DDD ASA exposure. c) Diclofenac users with more than 90 cumulative DDD ASA exposure. d) Ibuprofen users with less than 90 cumulative DDD ASA exposure. e) Ibuprofen users with more than 90 cumulative DDD ASA exposure. f) total time for all subjects from first NSAID prescription until endpoint in years. g) total immortal time is the total time for all subjects until they reached the 90 or 365 cumulative DDD threshold. h) median of cumulative defined daily dose NSAID exposure during follow up. i) Naproxen users with less than 90 cumulative DDD ASA exposure. j) Naproxen users with more than 90 cumulative DDD ASA exposure.

**Groups used in Time-dependent Cox Regression Analysis with 365DDD^a^ Threshold**

| Demographics 365DDD Threshold: | Diclofenac^b^ | Diclofenac + ASA^c^ |  | Ibuprofen^d^ | Ibuprofen + ASA^e^ |  |
| --- | --- | --- | --- | --- | --- | --- |
| Total Number: | 37.388 | 27.767 |  | 27.691 | 18.129 |  |
| Sex (Male %): | 35% | 40% |  | 28% | 36% |  |
| Follow-up time mean months (SD) | 78.8  (42.7) | 85.9  (41.3) |  | 73.0  (43.6) | 80.9  (42.8) |  |
| Total observation time (years)^f^ | 353.877 | 267.328 |  | 245.041 | 166.250 |  |
| Total Immortal time(years)^g^ | 108.504 | 68.548 |  | 76.568 | 44.030 |  |
| Mean age  (SD) | 71.8  (8.9) | 76.0  (9.2) |  | 69.8  (9.0) | 74.3  (9.4) |  |
| Age 50-55 (%) | 116 | 17 |  | 313 | 56 |  |
| Age 55-60 (%) | 1.346 | 284 |  | 2.152 | 452 |  |
| Age 60-65 (%) | 6.979 | 2.522 |  | 6.215 | 2.247 |  |
| Age 65-70 (%) | 9.059 | 4.762 |  | 6.969 | 3653 |  |
| Age 70-75 (%) | 7.812 | 5.844 |  | 5.075 | 3751 |  |
| Age 75-80 (%) | 4.776 | 4.722 |  | 2.818 | 2739 |  |
| Age > 80 (%) | 7.300 | 9.616 |  | 4.149 | 5239 |  |
| Median cumulative DDD exposure^h^ | 750 | 760 |  | 750 | 750 |  |
| Deaths (%) | 14.4% | 22.2% |  | 12.8% | 22.1% |  |
| PD Incidence number | 239 | 207 |  | 141 | 127 |  |
|  |  |  |  |  |  |  |
| Demographics 365DDD Threshold | **Naproxen^i^** | **Naproxen + ASA^j^** |  |  |  |  |
| Total Number | 27.959 | 18.722 |  |  |  |  |
| Sex (Male %): | 33% | 44% |  |  |  |  |
| Follow-up time mean months (SD) | 52.5  (44.5) | 64.8  (46.8) |  |  |  |  |
| Total observation time (years)^f^ | 169.285 | 133.082 |  |  |  |  |
| Total Immortal time(years)^g^ | 46.866 | 32.029 |  |  |  |  |
| Mean age  (SD) | 68.9  (9.2) | 74.9  (9.9) |  |  |  |  |
| Age 50-55 (%) | 1.160 | 174 |  |  |  |  |
| Age 55-60 (%) | 3.799 | 783 |  |  |  |  |
| Age 60-65 (%) | 5.486 | 1926 |  |  |  |  |
| Age 65-70 (%) | 5.582 | 3053 |  |  |  |  |
| Age 70-75 (%) | 4.705 | 3652 |  |  |  |  |
| Age 75-80 (%) | 2.944 | 3044 |  |  |  |  |
| Age>80 (%) | 4.283 | 6090 |  |  |  |  |
| Median cumulative DDD exposure^h^ | 780 | 800 |  |  |  |  |
| Deaths (%) | 10.0% | 19.3% |  |  |  |  |
| PD Incidence number | 100 | 85 |  |  |  |  |

a)Minimum of 365 cumulative defined daily dose of NSAID (diclofenac, ibuprofen or Naproxen) exposure during follow up. b) Diclofenac users with less than 90 cumulative DDD ASA exposure. c) Diclofenac users with more than 90 cumulative DDD ASA exposure. d) Ibuprofen users with less than 90 cumulative DDD ASA exposure. e) Ibuprofen users with more than 90 cumulative DDD ASA exposure. f) total time for all subjects from first NSAID prescription until endpoint in years. g) total immortal time is the total time for all subjects until they reached the 90 or 365 cumulative DDD threshold. h) median of cumulative defined daily dose NSAID exposure during follow up. i) Naproxen users with less than 90 cumulative DDD ASA exposure. j) Naproxen users with more than 90 cumulative DDD ASA exposure.
