# Supplementary material for: NSAID use is not associated with Parkinson’s disease incidence: A Norwegian Prescription Database study
Source: PLoS One. 2021 Sep 7;16(9):e0256602. doi: 10.1371/journal.pone.0256602 (PMC8423296; doi:10.1371/journal.pone.0256602)
Supplement: S2 Table — (DOCX) [file pone.0256602.s002.docx]

**S2 Table.**

**Demographics and descriptive statistics for Norwegian Prescription Registry**

**Groups used in Binary Logistic 5-year lag regression analysis with 90DDD^a^ Threshold**

| Demographics 90DDD Threshold: | Diclofenac^b^ | Diclofenac + ASA^c^ |  | Ibuprofen | Ibuprofen + ASA |  |
| --- | --- | --- | --- | --- | --- | --- |
| Total Number: | 81.960 | 44.417 |  | 50.013 | 26.231 |  |
| Sex (Male %): | 36% | 46% |  | 28% | 39% |  |
| Mean age  (SD) | 70.9  (8.9) | 76.6  (9.5) |  | 70.1  (9,0) | 75.7  (9.9) |  |
| Age 55-60 (%) | 4.693 | 740 |  | 4.032 | 766 |  |
| Age 60-65 (%) | 17.519 | 3.919 |  | 11.590 | 2.818 |  |
| Age 65-70 (%) | 19.845 | 6.792 |  | 12.247 | 4.390 |  |
| Age 70-75 (%) | 16.077 | 8.591 |  | 9.101 | 4.958 |  |
| Age 75-80 (%) | 9.530 | 7.498 |  | 5.162 | 4.018 |  |
| Age > 80 (%) | 14.296 | 16.877 |  | 7.881 | 9.281 |  |
| Median cumulative DDD exposure^d^ | 260 | 260 |  | 330 | 283 |  |
| Deaths (%) | 10.8% | 22.5% |  | 11% | 22.8% |  |
| Parkinson’s disease,  Incidence number | 380 | 269 |  | 192 | 155 |  |
|  |  |  |  |  |  |  |
| Demographics 90DDD Threshold | **Naproxen** | **Naproxen + ASA** |  |  |  |  |
| Total Number | 34.135 | 23.603 |  |  |  |  |
| Sex (Male %): | 36% | 47% |  |  |  |  |
| Mean age  (SD) | 70.1  (9.5) | 76.1  (9.9) |  |  |  |  |
| Age 55-60 (%) | 4.590 | 899 |  |  |  |  |
| Age 60-65 (%) | 7.891 | 2.261 |  |  |  |  |
| Age 65-70 (%) | 8.060 | 3.452 |  |  |  |  |
| Age 70-75 (%) | 6.681 | 4.293 |  |  |  |  |
| Age 75-80 (%) | 4.096 | 3.770 |  |  |  |  |
| Age>80 (%) | 6.621 | 8.928 |  |  |  |  |
| Median cumulative DDD exposure^d^ | 280 | 270 |  |  |  |  |
| Deaths (%) | 9.6% | 21.0% |  |  |  |  |
| Parkinson’s disease,  Incidence number | 141 | 95 |  |  |  |  |
|  |  |  |  |  |  |  |

a)Subjects who had 90 or more defined daily dose(DDD) of NSAID exposure during follow up, b) Group that had more than 90 DDD of diclofenac and less than 90 DDD of Acetylsalicyclic acid, c) Group that had more than 90 DDD of diclofenac and more than 365DDD of ASA. d) Median cumulative DDD exposure of NSAID during follow up

**Groups used in Binary Logistic 5-year lag regression analysis with 365DDD^a^ Threshold**

| Demographics 365DDD Threshold: | Diclofenac^b^ | Diclofenac + ASA^c^ |  | Ibuprofen | Ibuprofen + ASA |  |
| --- | --- | --- | --- | --- | --- | --- |
| Total Number: | 22.064 | 12.974 |  | 15.967 | 8159 |  |
| Sex (Male %): | 33% | 42% |  | 27% | 38% |  |
| Mean age  (SD) | 72.9  (9.2) | 77.7  (9.3) |  | 70.8  (9.1) | 75.6  (9.6) |  |
| Age 55-60 (%) | 689 | 138 |  | 1.060 | 201 |  |
| Age 60-65 (%) | 3.512 | 857 |  | 3.344 | 847 |  |
| Age 65-70 (%) | 4.900 | 1.726 |  | 3.990 | 1.423 |  |
| Age 70-75 (%) | 4.800 | 2.493 |  | 3.082 | 1.589 |  |
| Age 75-80 (%) | 3.036 | 2.261 |  | 1.715 | 1.280 |  |
| Age > 80 (%) | 5.127 | 5.499 |  | 2.776 | 2.819 |  |
| Median cumulative DDD exposure^d^ | 1025 | 910 |  | 1033 | 908 |  |
| Deaths (%) | 15% | 27.1% |  | 13.3% | 26% |  |
| PD Incidence number | 118 | 89 |  | 71 | 50 |  |
|  |  |  |  |  |  |  |
| Demographics 365DDD Threshold | **Naproxen** | **Naproxen + ASA** |  |  |  |  |
| Total Number | 11.969 | 7.656 |  |  |  |  |
| Sex (Male %): | 32% | 43% |  |  |  |  |
| Mean age  (SD) | 71.5  (9.7) | 76.9  (9.7) |  |  |  |  |
| Age 55-60 (%) | 1.110 | 226 |  |  |  |  |
| Age 60-65 (%) | 2.087 | 606 |  |  |  |  |
| Age 65-70 (%) | 2.543 | 1.041 |  |  |  |  |
| Age 70-75 (%) | 2.218 | 1.420 |  |  |  |  |
| Age 75-80 (%) | 1.451 | 1.268 |  |  |  |  |
| Age>80 (%) | 2.560 | 3.095 |  |  |  |  |
| Median cumulative DDD exposure^d^ | 1100 | 985 |  |  |  |  |
| Deaths (%) | 13% | 25% |  |  |  |  |
| PD Incidence number | 55 | 36 |  |  |  |  |
|  |  |  |  |  |  |  |
|  |  |  |  |  |  |  |
|  |  |  |  |  |  |  |
|  |  |  |  |  |  |  |

a)Subjects who had 365 or more defined daily dose(DDD) of NSAID exposure during follow up, b) Group that had more than 365 DDD of diclofenac and less than 90 DDD of Acetylsalicyclic acid, c) Group that had more than 365 DDD of diclofenac and more than 90DDD of ASA. d) Median cumulative DDD exposure of NSAID during follow up.
