# Supplementary material for: NSAID use is not associated with Parkinson’s disease incidence: A Norwegian Prescription Database study
Source: PLoS One. 2021 Sep 7;16(9):e0256602. doi: 10.1371/journal.pone.0256602 (PMC8423296; doi:10.1371/journal.pone.0256602)
Supplement: Attachment — Submitted filename: Rebuttal letter.docx [file pone.0256602.s003.docx]

Comments from reviewer 1:

The authors say that all subjects had an observation period of 12 or more months. The observation time is from 01/01/04 to 31/12/17. Would this mean that a person in group 1) could have used an average dose of NSAIDs of 0.0016 DDD/day (90 DDD over 156 months), while a person in group 2) could have used an average dose of 0.24 DDD/day (89 DDD over 12 months)? Wouldn’t this make the person in group 2) more exposed than the person in group 1)?

*Answer 1. A person with 90 DDD of cumulative exposure will have higher total exposure than someone who had 89 DDD cumulative exposure. Even though a person e.g. in group 2 could have higher average daily exposure during the follow-up time than another random person in the NSAID group (group 1), the cox regression model compares the Hazard ratio with respect to follow up time. Therefore, a person in the sporadic NSAID group (group 2) who had e.g. 30 months follow up time would be compared to those with 30 months follow-up time in the NSAID group (group 1), therefore, at each instance subjects in the NSAID group would always have higher cumulative NSAID exposure than someone in the sporadic group. As for the 5-year lag logistic regression analysis, by definition every subject in the NSAID group would have higher cumulative NSAID exposure. We have clarified that we talk about cumulative DDD use, this is incorporated throughout the manuscript.*

What is the thinking behind the “cut off” of 90 DDD of NSAIDs? Will this make the persons in group 2) sufficiently “unexposed”, so that their NSAID use will not protect dopamine neurons from dying? Opposite, will the persons using more than 90 DDD over the observation time be sufficiently exposed, so that their use of NSAIDs will confer neuroprotection?

*Answer 2. This is a good point. What defines a meaningful exposure of NSAID? Some studies divide NSAID exposure into groups, e.g. those who received few prescriptions and those who received multiple prescriptions (PMID: 21457177) or into ever or current use (PMID: 16606925). A similar study (PMID: 27471847) examining statin use with respect to PD used >28 DDD as threshold for classification for statin users. We believe that at least 90 DDD of cumulative use of NSAID constitutes a significant exposure. However, to reduce the risk that 90 DDD could be too low threshold, we have now included a parallel analysis with at least 365 DDD of cumulative NSAID use. The median of cumulative NSAID use in the 365 DDD groups was around 800-1000 DDD which constitutes a significant NSAID exposure.*

To elucidate the NSAID exposure in group 2) the authors could give data on the average cumulative NSAID dose before each person developed PD? This could be compared to the cumulative NSAID doses in persons in group 2) not developing PD.

*Answer 3. In Table 1-2 and supplementary 1-2 we have presented the median cumulative NSAID DDD exposure for all groups in both analyses. We have made it clear in the tables that this is cumulative NSAID used.*

Given that NSAIDs are seldom taken on a daily basis over a long period of time, but often in short periods when patients suffers from pain, I wonder how large was the gap in time between the last dose of NSAID and the development of PD. If this gap is very long (several months/years) do the authors believe that the NSAID use could be associated with development of PD? I would think that this could influence the chances of observing an association between the use NSAIDs and PD development.

*Answer 4. The hypothesis was that high cumulative exposure to NSAID should reduce risk of Parkinson’s Disease. The distribution of time from the last prescription to an endpoint (PD, death or end of observation) was for all NSAID groups heavily skewed toward shorter time. PD has a long prodromal phase (5-20 years), therefore it is the cumulative exposure of NSAID during the prodromal phase (or prior) that should theoretically be protective. If there was small gap in time it would imply that NSAID was used in the later stages of the prodromal phase, whereas a large gap would imply that NSAID was used in the early prodromal phase or prior. One could argue that either could be more/less beneficial. It is not known if a potential neuroprotective agent should be used in later stages of a prodromal phase or in the early stages of the prodromal phase. This retrospective study cannot address this question. A sensitivity analysis with subgrouping of gap of time from last NSAID to an endpoint would bias the shorter gap group toward higher PD incidence and those with longer gap toward lower PD incidence. This is because those NSAID users with the longest follow-up time are more likely to meet the end of observation time (31.12.2017) rather having died or had incident PD (the two other endpoints). Conversely is also true, those with the shortest follow-up time are more likely to have died or become incident PD.*

As mentioned above I wonder about the comparison between group 1) and 2); is group 2) a control? Would it not be better to compare PD incidence in the NSAID exposed groups to the PD incidence in the Norwegian population not taking any NSAIDs?

*Answer 5. Yes, group 2 is a control. We compare those subjects who used little or negligible cumulative doses of NSAID to who used a high cumulative dose of NSAID. All NSAID groups are in general very large, ranging from 300.000 subjects to 20.000 subjects. The groups are thus very likely to be representative of the general population and so large that it is unlikely that there should be any sample biases. Furthermore as seen in table 1-2 and supplementary table 1-2, the follow up time, age and sex demographics is very similar across groups. To strengthen the argument that the sporadic NSAID group (group 2) and ASA group (group 4) (the control groups) do indeed have PD incidence representative for the general population, we have included a new table to the results with age-specific incidence rates of the Sporadic NSAID group (group 2) and the ASA group (group 4). The age-specific incidences in the control groups are similar to earlier literature reporting on age-specific incidence in PD. We have amended the result section and discussion to highlight this.*

Even though recent meta-analyses suggest that NSAIDs do not protect dopamine neurons from dying in PD, there are some discrepancies as described in the MS, in particular related to ibuprofen. Thus, would it not increase the validity of the author’s conclusion if they examined the relation between the use of ibuprofen and PD incidence separately? I also think it would of interest to the readers if the authors could present the main subtype of NSAIDs used by the persons in the study (also see below).

*Answer 6. This is a good point. We have performed secondary analysis where we have performed time-dependent Cox regression analysis and 5-year lag binary logistic regression analysis for the subgroup of diclofenac, ibuprofen and naproxen, the most commonly prescribed NSAID in the Norwegian population. None of the specific NSAIDs were associated with lower PD incidence. Result section and discussion has been expanded to incorporate these results.*

Defined daily dose (DDD) should be explained. The authors should also refer to a site where the readers could find information about the dose in mg/g, which corresponds to 1 DDD (e.g. WHO Collaborating Centre for Drug Statistics Methodology, ATC classiﬁcation index with DDDs, 2020. Oslo, Norway), or the authors could make a table in which the DDD of the main NSAIDs investigated in the study is given.

*Answer 7. We have added a definition of DDD as stated by the WHO Collaborating Centre for Drug Statistics Methodology and referenced this centre in the method section as well as to all the legend of all tables.*

Immortal time bias should be explained. The discussion of the impact of correction for immortal time bias in the Discussion is quite difficult to follow for generalists and should be rewritten to make the points more clear.

*Answer 8. Thank you for highlighting this point. We have included the person-years of immortal time as well as the total follow up time for each group to table 1 and supplementary table 1.We have also expanded the discussion of immortal time.*

In Methods, p.6, l. 18-20 the authors say that “for the overall analysis the observation period started 12 months after the first dispensed prescription.”. The authors should explain why this was done. They should explain how only incident PD cases were included in the study.

*Answer 9. This was to ensure a minimum exposure time from the first prescription. We have added this to the method section.*

The authors should explain the rationale for doing “the 5-year lag analysis”.

*Answer 10. It was done under the assumption there could be a potential latency period from exposure to outcome (PD incidence). Secondary we wanted to include an analysis without immortal time in case immortal time would bias the results. We have added this to the method section.*

In Methods, p. 7, l. 11-13 the authors say that “the age and sex covariates had a significant contribution to the model and satisfied the proportional hazard assumption which was verified by examining the DFBETA residuals against time and log(-log[PD incident]) versus log(time) curved ”. The meaning of this is unclear and DFBETA is not defined.

*Answer 11. There are several ways to verify the proportional hazard assumption to the cox regression model, examining residuals such as Schoenfeld, Cox-snell or by looking at the log-log survival curves. The DFBETA is used to assess an individual’s impact on the model. Given the model and covariates (sex and age), the DFBETA is superfluous to mention as no single individual would have a significant impact. We have therefore simplified this and report in the statistical method section that we verified that the proportional hazard assumptions were met by inspecting the log(-log(survival)) curves.*

Some co-morbidities, for which NSAIDs could be subscribed, may be related to PD development. Would the authors consider to correct for such co-morbidities? For instance inflammatory bowel disease seems to be associated with increased risk of PD (e.g. Villumsen M Gut 2019; Weimers P Inflamm Bowel Dis 2019). The same is true for diabetes ( e.g. Yue X Medicine 2016). If this is the case, could it mask the observed effect of NSAIDs on PD incidence in this study? Rheumatoid arthritis (RA) is probably related to a decreased risk of PD (Li C NPJ Parkinsons Dis 2021) and would not explain the results, unless the number of persons with RA was larger in the “control” groups in the study.

*Answer 12. It is a limitation of our study that it is not possible to correct for these co-morbidities using the prescription registry at hand. We addressed this concern in the discussion (p13). However due to the very large groups analyzed, it is unlikely that there should be an uneven distribution of co-morbidities that are not correlated to NSAID use. RA is a co-morbidity that we could expect to have a higher frequency in the high cumulative NSAID exposure groups, but this should favor decreased incidence of PD in the NSAID groups, which we did not find. We have amended the discussion to highlight this point.*

In Methods, p. 5, l. 23 the authors say that NSIADs were identified by the following ATC Codes; M01A* and S01BC*. The latter is eye preparations. The authors should clarify.

*Answer 13. We thank the reviewer for detecting this error. S01BC* is indeed an eye preparation and does not have corresponding DDD. As stated in the original draft, all NSAID medication that were not orally administered were excluded. We have amended the draft and removed “S01BC*”. We have also checked the incidence of occurring NSAID prescriptions and only prescription with ATC code M01A* is indeed included.*

One finding was that there was a 17% higher hazard ratio for PD among NSAID users compared to those who did not use NSAID (although not reproduced in another regression model). Could it be that the effect of NSAIDs in PD cases, who have progressed into the motor phase, is to inhibit clearance of increased level of extracellular alpha-synuclein by microglia, thereby worsening the PD course?

*Answer 14. We have expanded the results section. As before, we found that there was an increased incidence of PD in the NSAID group (1) compared to the sporadic NSAID group(2) for both the 90 and 365 DDD threshold analysis as well as the binary logistic analysis. The NSAID plus ASA(3) compared to the ASA group(4) was however not significant. Whether the NSAID group(1) has a true higher PD incidence is therefore inconclusive. There is however no indication of lower PD incidence. As to whether NSAID use could affect alpha-synuclein and microglia, it is an interesting idea, however in the light of the results which show overwhelming support for no significant difference increase of PD among NSAID users, we feel that we cannot speculate on whether NSAID does indeed cause an increased risk of PD.*

In Introduction, p. 4, l. 14 the references cited (8 and 9) must be wrong.

*Answer 15. Would like to thank the reviewer for bringing this to our attention. There was an error due to the reference manager used. The reference list has been updated and verified and some new references has also been added to the discussion to reflect the amendments made. The reference list is now up to date.*

In Discussion, p.11, l. 7-8: how could “the minimal stratification” both be a strength and a weakness?

*Answer 16. We agree that this was unclear. We have rewritten this paragraph in the discussion.*

I do not completely agree with the basic idea of assimilating neuroinflammation and systemic inflammation; consequently a study on NSAIDs may be too simplistic.

*Answer 17. We agree that this may indeed be too simplistic. However, as the literature on NSAID use and PD incidence has showed conflicting results, our rationale was to examine this hypothesis in a very large population-based cohort. If there indeed was a true neuroprotective effect from NSAID use, this study should be sufficiently powered to detect an effect. Given the very large sample size available through the prescription registry, we think our results are a significant contribution to the literature and the debate as to whether NSAID use influences risk of PD.*

I would also look at the other side of the coin: not only the use of NSAIDs to reduce inflammation, but also the possibility that those who use NSAIDs probably start from a more pronounced inflammatory condition.

*Answer 18. This could very well be the case, but NSAID use could also very likely be due to a pain condition. Assuming however that the NSAID users did indeed have a more pronounced inflammatory condition, one would expect them to have an increased risk of PD . In the literature on NSAID use and risk of PD, the findings have either shown non-significant or lower risk associated with NSAID use. This study was not designed to identify the mechanism behind a protective or detrimental effect of NSAID use , but whether NSAID use is associated with neuroprotection, as has been suggested in the literature.*

Does the Norwegian Prescribing Database take into account the whole population? In other words, were people who did not receive any prescriptions in their life (until the authors' research) also included in the Sporadic group?

*Answer 19. The Norwegian Prescription Database do take into account the whole population. However, to be able to make groups comparable, low/sporadic NSAID users were compared to those with a significant cumulative NSAID exposure. Please see Answer 5 for a more thorough explanation.*
